# Predictors of In-Hospital Mortality Among Paediatric Snakebite Patients in Northeastern Nigeria: A Survival Analysis Study

**DOI:** 10.12688/wellcomeopenres.26196.1

**Published:** 2026-04-28

**Authors:** Aashna Uppal, Nicholas Amani Hamman, Nuhu Mohammed, Abigail Micah Umar, Surya M, Eric Nyarko, Suraj Abdulkarim, Trudie Lang, Joshua Abubakar Difa

**Affiliations:** 1 The Global Health Network, Nuffield Department of Medicine, University of Oxford, Oxford, England, OX27SG, UK; 2 Snakebite Treatment and Research Hospital, Kaltungo, Gombe, Nigeria; 3 Department of Community Medicine and Public Health, Gombe State University, Gombe, Gombe, Nigeria; 4 Sri Sai Ram Institute of Technology, Chennai, Tamil Nadu, India; 5 Department of Statistics and Actuarial Science, University of Ghana College of Basic and Applied Sciences, Accra, Greater Accra Region, Ghana; 6 Gombe State Ministry of Health, Gombe, Gombe, Nigeria

**Keywords:** Paediatric Health, Mortality, Snakebites, Low-Resource Settings, Risk Factors, Neglected Tropical Diseases

## Abstract

Paediatric patients of snakebite envenoming are vulnerable to severe complications due smaller body size and dependence on adults for timely medical attention. However, there is limited literature on risk factors for mortality in pediatric populations, particularly within rural Nigerian settings, where snakebite envenoming is prevalent.

A retrospective study was conducted using medical records at the Snakebite Treatment and Research Hospital (SBTRH) in Northeastern Nigeria, including paediatric patients aged 0 through 17 years who presented with a snakebite from January through December 2024. We estimated cumulative survival probability over time using Kaplan-Meier curves and identified predictors of mortality using multivariable Cox proportional hazards models.

SBTRH managed 2,192 snakebite patients in 2024, out of which 723 paediatric patients were included in analyses; 702 of these patients were discharged alive, while 21 died in hospital. Those who took four hours or more to arrive at the hospital after being bitten were more likely to die in hospital compared to those who arrived in less than four hours (hazard ratio (HR) = 5.87, 95% CI = 1.15-29.95). Further, those who were not given antivenom (Echitab) were more likely to die in hospital than those who were given antivenom (HR = 10.07, 95% CI = 3.89-26.07).

Aligning with other studies on poor outcomes following snakebite, this study identifies delayed hospital presentation and lack of antivenom administration among key predictors of in-hospital mortality among paediatric snakebite patients in Northeastern Nigeria. These findings reiterate a need for strengthening antivenom supply chains and implementing targeted educational campaigns.

## Plain Language summary

Children who are bitten by venomous snakes are vulnerable to poor outcomes because of their small bodies and dependence on adults to seek timely medical attention. In Nigeria, venomous snakebites among children are common. At a large snakebite hospital in Northeastern Nigeria, the data of 723 children bitten by venomous snakes was analyzed. Among these 723 children, 21 died in hospital. Those who took longer than four hours to arrive at hospital had increased risk of death, and those who received antivenom, which is the mainstay treatment for venomous snakebites, had decreased risk of death. These findings suggest that it is vital to educate the community on the importance of coming to hospital early, as well as advocate for free antivenom provision where it is needed most.

## Introduction

Snakebite envenoming (SBE) persists as a neglected tropical disease, inflicting profound suffering and loss of life, especially among the rural and underserved populations of Sub-Saharan Africa (SSA) ( Ayesiga et al., 2025; Gopal et al., 2023; Habib, 2013; Matlani, 2024). The World Health Organization (WHO) estimates that globally, snakebites affect 5.4 million people annually, of which about 2.7 million people suffer from envenomation, and approximately 81,000 to 138,000 die ( Gutierrez et al., 2017). This translates to approximately 10 snakebites per minute, with around 5 resulting in envenomation, and one death occurring every 4 to 7 minutes. Nigeria has the highest incidence of snakebite in SSA, with most cases occurring in the northern savannah and Benue Valley regions, where farming, herding, and rural lifestyles increase the risk of snake encounters ( Gutiérrez et al., 2021; Habib et al., 2001; Pugh and Theakston, 1980). Despite this burden, snakebite remains under-reported and inadequately addressed by health systems, particularly regarding its impact on children ( Naik and Alexander, 2025; Silvestri et al., 2024).

In northeastern Nigeria, the saw-scaled or carpet viper **(** *Echis romani*), the puff adder ( *Bitis arietans*), and cobras ( *Naja nigricollis*) are the three predominant venomous snake species reported, collectively responsible for the majority of SBE cases in the region ( Gutiérrez et al., 2021; Hamman et al., 2025c). Among these, the carpet viper is the most frequently implicated and is associated with severe clinical outcomes including coagulopathy, systemic hemorrhage, hypovolemic shock, tissue necrosis, and if left untreated, has a case fatality rate of 10-20% ( Warrell et al., 1977) **.** The puff adders and cobras contribute significantly as well, with envenomation often resulting in cytotoxic and neurotoxic effects ( Kakaria et al., 2014; Niiteta, 2022; Yunin et al., 2023).

Children may be vulnerable to severe complications following snakebite due to their smaller body size, lower physiological reserves, and dependence on adults for timely medical attention, which often results in delayed access to care; however, differences in features of envenoming between children and adults are highly species-specific ( Iliyasu et al., 2023b; Sankar et al., 2013; Zdenek et al., 2023) **.** Risk factors such as delayed hospital presentation **,** late or insufficient antivenom administration **,** the species of snake involved **,** the anatomical site and time of the bite **,** and the presence of systemic complications (for example, shock, coagulopathy, or central nervous system involvement) have been associated with mortality in studies including adult patients ( Abdullahi et al., 2022; Hamman et al., 2025c, 2025d; Wood, 2023). However, literature on these risk factors in pediatric populations remains limited. This gap highlights the a need for context-specific research that can capture the temporal dynamics of snakebite mortality in children.

Further, although snakebite is recognised as a public health problem in West Africa, few studies from Nigeria or the broader region have examined paediatric outcomes using time-to-event analysis. Existing literature, mostly cross sectional or descriptive ( Abdullahi et al., 2022; Habib, 2013; Hamman et al., 2025c, 2025d, 2025b; Igawe et al., 2020; Iliyasu et al., 2023b; Lugga and Bawa-Sani, 2025; Ndu et al., 2018; Pugh and Theakston, 1980; Sani et al., 2013; Sankar et al., 2013; Wood, 2023; Zdenek et al., 2023), has documented the incidence, case fatality, antivenom use, and other static risk factors for mortality in children, adults and pregnant women, but has not explored how rapidly death occurs after envenomation or treatment, through time-to-event analysis, among children. 

The present study addresses this evidence gap using survival-analysis methods (Kaplan–Meier and Cox proportional hazards models), which allow estimation of time-dependent mortality and hazard rates ( Lee, 2023). We previously analysed paediatric snakebites in Northeastern Nigeria using the same hospital based dataset as this report ( Hamman et al., 2025a), providing the largest single-centre description of disease burden and predictors of poor recovery (both death and complications) in the region. However, that study was limited to descriptive and logistic regression analyses and could not assess mortality as a time-dependent outcome or determine which factors elevate the risk of death once a child is admitted to hospital. Consequently, important temporal dimensions of risk remained unexplored.

Accordingly, this study conducts a one-year retrospective survival analysis of paediatric snakebite patients admitted to the Snakebite Treatment and Research Hospital (SBTRH) in Gombe State, Northeastern Nigeria. By applying a new analytic framework to a previously described cohort, the study ensures transparency, avoids redundancy, and generates actionable evidence on time-dependent mortality patterns, insights currently lacking in both our prior work and the broader West African snakebite literature.

## Materials and methods

### Study setting and population

The current study was a retrospective study conducted at SBTRH in Kaltungo, Gombe State, Northeastern Nigeria. SBTRH is the largest hospital of its kind, in terms of the number of snakebite patients, in SSA. Individuals present to SBTRH from within Gombe State, from neighbouring states in Northeastern Nigeria, and from neighbouring countries like Chad and Cameroon. Accordingly, SBTRH is adequately representative of the snakebite epidemiology observed in Northeastern Nigeria.

The sample size of patients was not predetermined, but rather, was based on the following inclusion criteria: paediatric patients aged 0 through 17 who presented at SBTRH with a snakebite from January through December 2024, who did not abscond or leave against medical advice. This age range was chosen to match studies previously conducted on paediatric populations in this region ( Hamman et al., 2025a; Lugga and Bawa-Sani, 2025; Ndu et al., 2018). However, because some studies define paediatric patients as under five years of age or under 15 years of age, we included age groups in our analyses as well (0-4, 5-9, 10-14, and 15-17).

### Data collection and analysis

Patient folders were accessed by trained medical staff at SBTRH (authors NAH, NM, AMU) on 1 May 2025; AMU conducted initial abstraction of patient data, which was then cross-checked by NAH and NM. Any conflicts were resolved by NAH (chief medical director). These staff members could identify individual patients during data collection but ensured that no identifiable data was documented for the purposes of research. Specifically, the following information was documented in a Microsoft Excel file from each included patient folder: month of snakebite occurrence, patient age, patient sex, patient state of origin, patient occupation, anatomical site of snakebite, species of snake, number of antivenom vials used, cost of antivenom (free vs. paid), number of hours between snakebite and presentation to hospital, number of hours between presentation to hospital and antivenom administration, duration of hospital stay, and patient outcome (death versus discharged alive). The outcome definition was unique from our previous study ( Hamman et al., 2025a), in that we focused solely on death in hospital. These variables were chosen as they tend to have complete information recorded for all patients; since 2019, record-keeping at SBTRH has been standardized. Of note, species identification relies on the patient either bringing in the dead snake (which upwards of 60% of patients do) or identifying the snake that bit them using a poster in the admissions room, corroborated by observed symptoms. Any missing data was left as-is, with the label “not reported” or “unidentifiable”.

Patient data was then cleaned and analyzed using R software (version 4.3.1). We performed a three-stage analysis. First, we used descriptive statistics (proportions, medians, and interquartile ranges) to summarize patient characteristics grouped by outcome at the end of hospitalization (death versus discharge alive). The student’s t-test was used for continuous variables, Fisher’s exact test was used for categorical variables with cell counts less than five, and Pearson’s Chi-squared test was used for categorical variables with cell counts greater or equal to five, to summarize differences in patient characteristics between those who died in hospital and those who were discharged alive. Variables for which p-values for the corresponding statistical test were less than 0.05 were considered to be significantly associated with patient mortality in descriptive analyses. Further, for each variable, any categories with less than five patients were grouped into a generic category labelled “Other”.

To prepare for the following analyses, we created a subset of patients by excluding any patients with missing information and patients belonging to “Other” categories. For variable categories with zero cell counts, we made logical groups where possible (for example, grouping individual months of presentation into seasons), and where that wasn’t possible, we excluded that variable from further analysis.

Second, we estimated cumulative survival probability over time using Kaplan-Meier curves, which are non-parametric and make no distributional assumptions about the data. Given that this analysis was exploratory, non-parametric methods were deemed appropriate. Accordingly, we estimated overall survival for the paediatric cohort, as well as group-wise survival for each variable, separately. Log-rank tests were used to calculate p-values. Variables that had p-values less than 0.05 in these analyses were considered to be significantly associated with better or worse survival over time. Further, we considered three distinct time-to-event measures: (1) days between snakebite and hospital discharge, (2) days between hospital arrival and discharge, and (3) days between antivenom administration and discharge. For the third time-to-event measure, only patients who received antivenom were included in the analyses.

Third, we identified predictors of death using multivariable Cox proportional hazards models. The Cox proportional hazards method is semi-parametric. When building multivariable models, we included only variables that did not violate the proportional hazards assumption. Further, if variables demonstrated high degrees of multicollinearity, separate models were created including each of the collinear variables. Through this process, adjusted hazard ratios were produced. Hazard ratios with 95% confidence intervals (CIs) that did not overlap the number one indicated a significant increase (above one) or decrease (below one) in mortality risk. Similar to the log-rank tests, Cox proportional hazards modelling was performed for each of the three time-to-event measures.

### Public and patient involvement

Ethical clearance was obtained from the Gombe State Ministry of Health (Ref: MOH/ADM/621/V.1/497), and necessary approvals were obtained from the Gombe State Hospital Services Management Board. This study was part of a larger project to generate evidence from existing health data at SBTRH and abided by the principles outlined in the Declaration of Helsinki. Through this approval process, the requirement for individual patient consent was waived. As well, since the data was retrospective, the public and patients were not involved in the design or undertaking of this study. However, key stakeholders - namely, hospital management officials, community leaders and snakebite advocates - were engaged to disseminate study results.

## Results

SBTRH managed 2,192 snakebite patients between 1 January and 31 December 2024, out of which 761 were under the age of 18; 23 paediatric patients had blank medical records and 15 absconded or left against medical advice. Of the 723 paediatric patients included in analyses, 702 were discharged alive, while 21 died in hospital. The characteristics of these patients are described in Table 1. Of note, the only two variables missing information were snake species and time to antivenom administration.

**Table 1.** Sociodemographic and clinical characteristics of included patients.

Variable	Overall, N = 723 *^1^*	Patient outcome at discharge		p-value *^2^*
		Dead, N = 21 *^1^*	Alive, N = 702 *^1^*	
**Patient Demographics**				
**Age**	12.0 (8.0, 15.0)	14.0 (8.0, 16.0)	11.5 (8.0, 15.0)	0.209
**Age Group**				0.171
10 to 14	300	7 (2.3)	293 (98%)	
5 to 9	189	3 (1.6)	186 (98%)	
15 to 17	185	8 (4.3)	177 (96%)	
0 to 4	49	3 (6.1)	46 (94)	
**Sex**				0.978
Male	480	14 (2.9)	466 (97)	
Female	243	7 (2.9)	236 (97)	
**State of Origin**				0.024
Gombe	364	9 (2.5)	355 (98)	
Taraba	122	1 (0.8)	121 (99)	
Adamawa	90	4 (4.4)	86 (96)	
Bauchi	67	1 (1.5)	66 (99)	
Borno	50	6 (12)	44 (88)	
Yobe	26	0 (0)	26 (100)	
Other *^4^*	4	0 (0)	4 (100)	
**Occupation**				0.499
Under Care *^5^*	413	10 (2.4)	403 (98)	
Farmer	143	7 (4.9)	136 (95)	
Student	141	4 (2.8)	137 (97)	
House Wife	21	0 (0)	21 (100)	
Other *^4^*	5	0 (0)	5 (100)	
**Circumstances of Snakebite**				
**Month of Snakebite Occurrence**				
April	102	3 (2.9)	99 (97)	
March	81	0 (0)	81 (100)	
August	73	4 (5.5)	69 (95)	
October	72	4 (5.6)	68 (94)	
July	67	3 (4.5)	64 (96)	
May	64	0 (0%)	64 (100)	
June	59	1 (1.7)	58 (98)	
September	58	3 (5.2)	55 (95)	
February	47	1 (2.1)	46 (98)	
November	39	2 (5.1)	37 (95)	
January	31	0 (0)	31 (100)	
December	30	0 (0)	30 (100)	
**Hours Between Bite and Hospitalization** *^3^*				0.012
≥ 4 hours	468	19 (4.1)	449 (96)	
< 4 hours	255	2 (0.8)	253 (99)	
**Site of Snakebite**				0.361
Upper Limb	434	10 (2.3)	424 (98)	
Lower Limb	284	11 (3.9)	273 (96)	
Other *^4^*	5	0 (0)	5 (100)	
**Snake Species**				0.831
Carpet Viper	559	16 (2.9)	543 (97)	
Unidentified	157	5 (3.2)	152 (97)	
Other *^4^*	7	0 (0)	7 (100)	
**Treatment**				
**Antivenom Dose**				<0.001
1 vial	512	11 (2.1)	501 (98)	
0 vials	112	10 (8.9)	102 (91)	
≥ 2 vials	99	0 (0)	99 (100)	
**Hours Between Hospitalization and Antivenom Administration** *^3^*				0.002
≥ 1 hour	491	10 (2.0)	481 (98)	
None given	112	10 (8.9)	102 (91)	
< 1 hour	87	1 (1.1)	86 (99)	
Not Reported	33	0	33	
**Antivenom Cost**				<0.001
Free	351	4 (1.1)	347 (99)	
Paid *^6^*	260	7 (2.7)	253 (97)	
None given	112	10 (8.9)	102 (91)	

^1^ n (%) or median (interquartile range).

^2^ Student’s t-test for continuous variables; Fisher’s exact test for categorical variables (cell counts less than five); Pearson’s Chi-squared test for categorical variables (cell counts greater or equal to five). For one variable (month of presentation), p-values could not be calculated due to low cell counts.

^3^ The median number of hours between bite and hospitalization is 4, and the median number of hours between hospitalization and antivenom administration is 1.

^4^ Other values for state of origin are Cameroun and Plateau, for occupation are applicant and business, for site of snakebite are back and neck, and for snake species are cobra, night adder, and puff adder.

^5^ Under care refers to patients 16 years of age and under who are neither working nor in school.

^6^ Paid means the patient paid either for a full or partial dose.

The median age of patients was 12 years old (interquartile range 8 to 15 years), with the largest proportion of patients belonging to the 10 to 14 year age group (n = 300, 41%). Two-thirds of patients were male (n = 480, 66%) and took four hours or more to arrive at the hospital (n = 468, 65%). Most patients were prescribed a single dose (single vial) of Echitab antivenom (n = 512, 71%) one hour or more after hospital admission (n = 491, 68%). Of note, 112 patients did not receive antivenom due to a lack of availability at the time. Approximately half of the cohort received antivenom free of charge (n = 351, 49%) and arrived at the hospital from within Gombe state (n = 364, 50%). Snakebites demonstrated a seasonal trend, with one-quarter of the entire year’s bites occurring in March and April (n = 183, 25%), at the onset of the rainy season. More than half of the cohort was under care (n = 413, 57%), which refers to patients aged 16 years and under who are neither working nor in school. Most bites occurred in the upper limb (n = 434, 60%) (a finding previously discussed in our other study ( Hamman et al., 2025a)), and the majority of patients were bitten by carpet vipers (n = 559, 77%).

There were notable differences in patient characteristics between those who died in the hospital and those who were discharged alive. The number of hours between bite and hospitalization (p = 0.012), antivenom dose (p < 0.001), hours between hospitalization and antivenom administration (p = 0.002), antivenom cost (p < 0.001), and state of origin (p = 0.024) were all significantly associated with survival.

**Figure 1.**
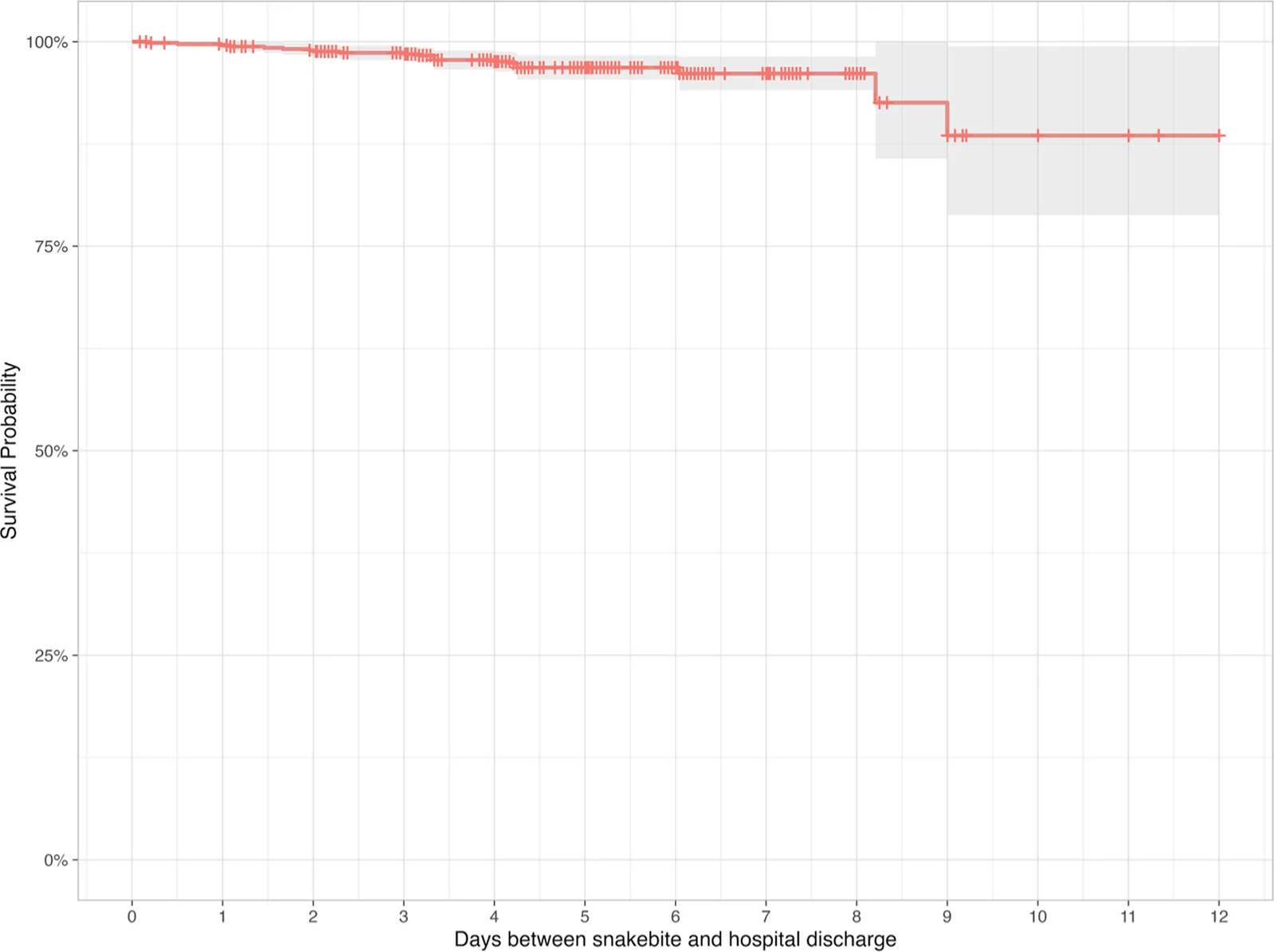
Overall survival probability from snakebite to hospital discharge.

Figure 1 illustrates the overall survival curve for a subset of patients from this cohort with no missing information and variable cell counts above zero (n = 677). The survival probability remained high throughout the full time period (days between snakebite and hospital discharge), decreasing from 100% to just above 88%. Results for overall survival from hospital admission to discharge and from antivenom administration to discharge (Figures S1, S2) were qualitatively similar, albeit with higher survival probabilities.

Table 2 shows log-rank estimates and p-values for covariates associated with survival in univariate analyses. When considering the full time period (days between snakebite and hospital discharge), as well as the time from hospital admission to discharge, antivenom administration (p < 0.001), antivenom cost (p < 0.001), and hours between hospital admission and antivenom administration (p < 0.001) were all significantly associated with cumulative survival probability: consistently, those who were given antivenom had better overall survival than those who did not receive antivenom. The hours between bite and hospitalization also demonstrated a significant association with cumulative survival probability (p = 0.043 for full time period; p = 0.014 for time between hospital admission and discharge); those who arrived four hours or more after being bitten had a worse survival rate than those who arrived in less than four hours. Finally, seasonality demonstrated a significant association with cumulative survival probability (p = 0.045 for full time period; p = 0.049 for time between hospital admission and discharge), with snakebites occurring during the rainy season having worse survival than those occurring during the dry season. Other covariates did not demonstrate a significant association with the cumulative survival probability between snakebite and hospital discharge, nor between hospital admission and discharge. As well, no covariates demonstrated a significant association with cumulative survival probability from antivenom administration to hospital discharge; notably, this analysis only included those who received antivenom.

**Table 2.** Log rank tests of differences in survival (Univariate).

Variable	Categories	Time from snakebite to hospital discharge			Time from hospital admission to discharge			Time from antivenom administration to hospital discharge **		
		Survival *	χ2	p	Survival *	χ2	p	Survival *	χ2	p
Age Group	0 to 4	Worse	5.056	0.168	Worse	4.588	0.205	Better	6.918	0.075
	5 to 9	Better			Better			Better		
	10 to 14	Better			Better			Worse		
	15 to 17	Worse			Worse			Worse		
Sex	Female	Better	0.013	0.908	Worse	0	0.995	Better	0.179	0.672
	Male	Worse			Better			Worse		
State of Origin	Within Gombe	Better	0.044	0.833	Better	0.439	0.508	Better	0.816	0.366
	Outside of Gombe	Worse			Worse			Worse		
Occupation	Under Care	Better	2.567	0.277	Better	2.484	0.289	Better	4.859	0.088
	Farmer	Worse			Worse			Worse		
	Other	Better			Better			Better		
Season of Snakebite Occurrence	Dry Season	Better	4.006	0.045	Better	3.877	0.049	Better	1.515	0.218
	Rainy Season	Worse			Worse			Worse		
Hours Between Bite and Hospitalization	< 4 hours	Better	4.094	0.043	Better	5.987	0.014	Better	3.242	0.072
	≥ 4 hours	Worse			Worse			Worse		
Site of Snakebite	Upper Limb	Better	0.879	0.348	Better	1.369	0.242	Better	2.582	0.108
	Lower Limb	Worse			Worse			Worse		
Snake Species	Carpet Viper (Echis Romani)	Worse	0.003	0.956	Better	0.014	0.905	Worse	0.938	0.333
	Unidentifiable	Better			Worse			Better		
Antivenom Given	Yes	Better	34.73	<0.001	Better	25.14	<0.001			
	No	Worse			Worse					
Hours Between Hospitalization and Antivenom Administration	No Antivenom Given	Worse	35	<0.001	Worse	25.41	<0.001		0.426	0.514
	< 1 hour	Better			Better			Better		
	≥ 1 hour	Better			Better			Worse		
Antivenom Cost	No Antivenom Given	Worse	35.43	<0.001	Worse	26.61	<0.001		3.13	0.077
	Free	Better			Better			Better		
	Paid (for Full or Partial Dose)	Better			Better			Worse		

* Survival is compared to other categories of that variable, rather than a reference category. Better survival is defined as observed deaths being less than estimated deaths in that category (as per the log-rank test); worse survival is defined as observed deaths being greater than estimated deaths in that category.

** These analyses only consider patients who received antivenom.

**Figure 2.**
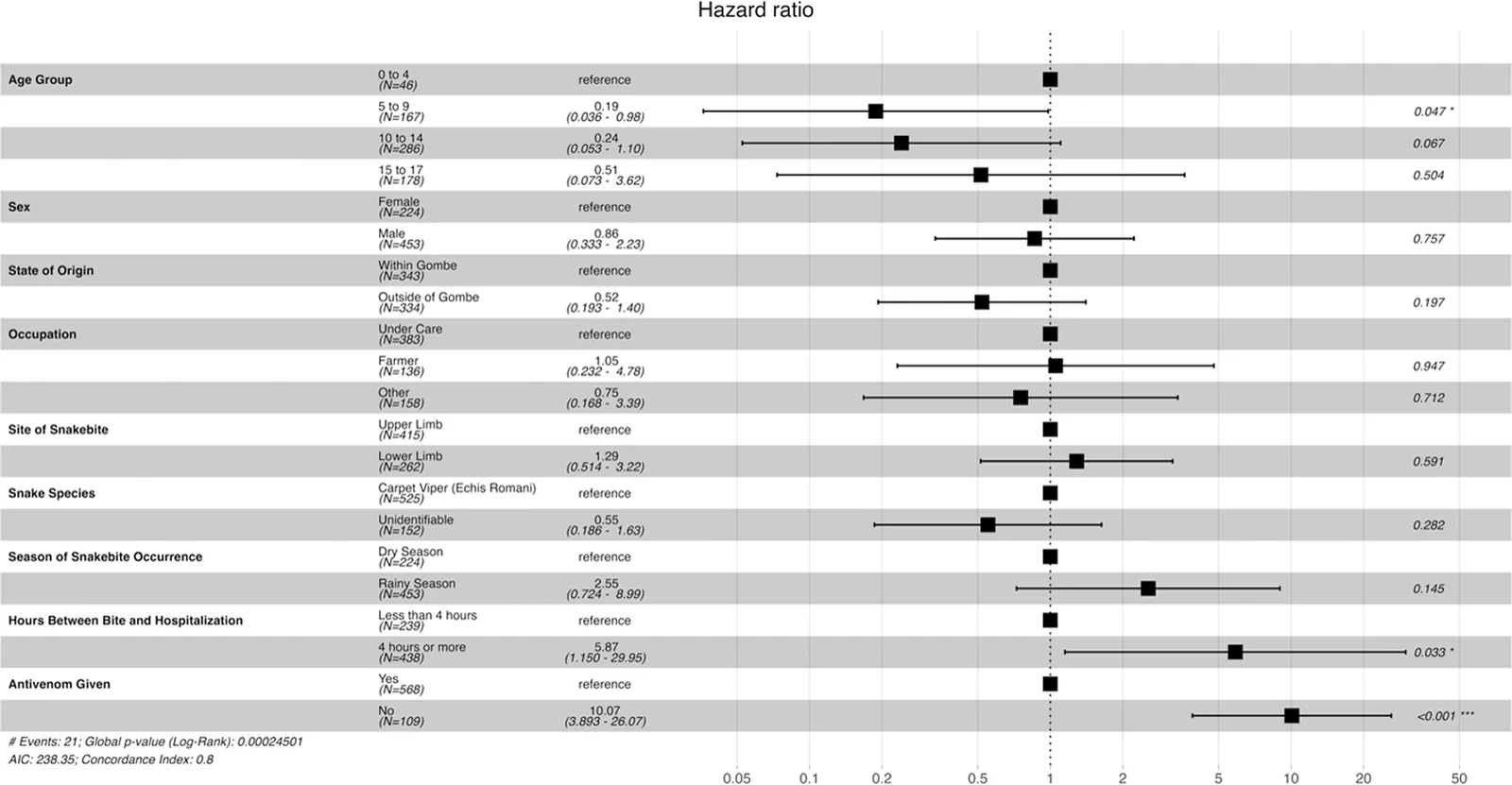
Cox proportional hazards model for survival between snakebite and hospital discharge (antivenom administration).

**Figure 3.**
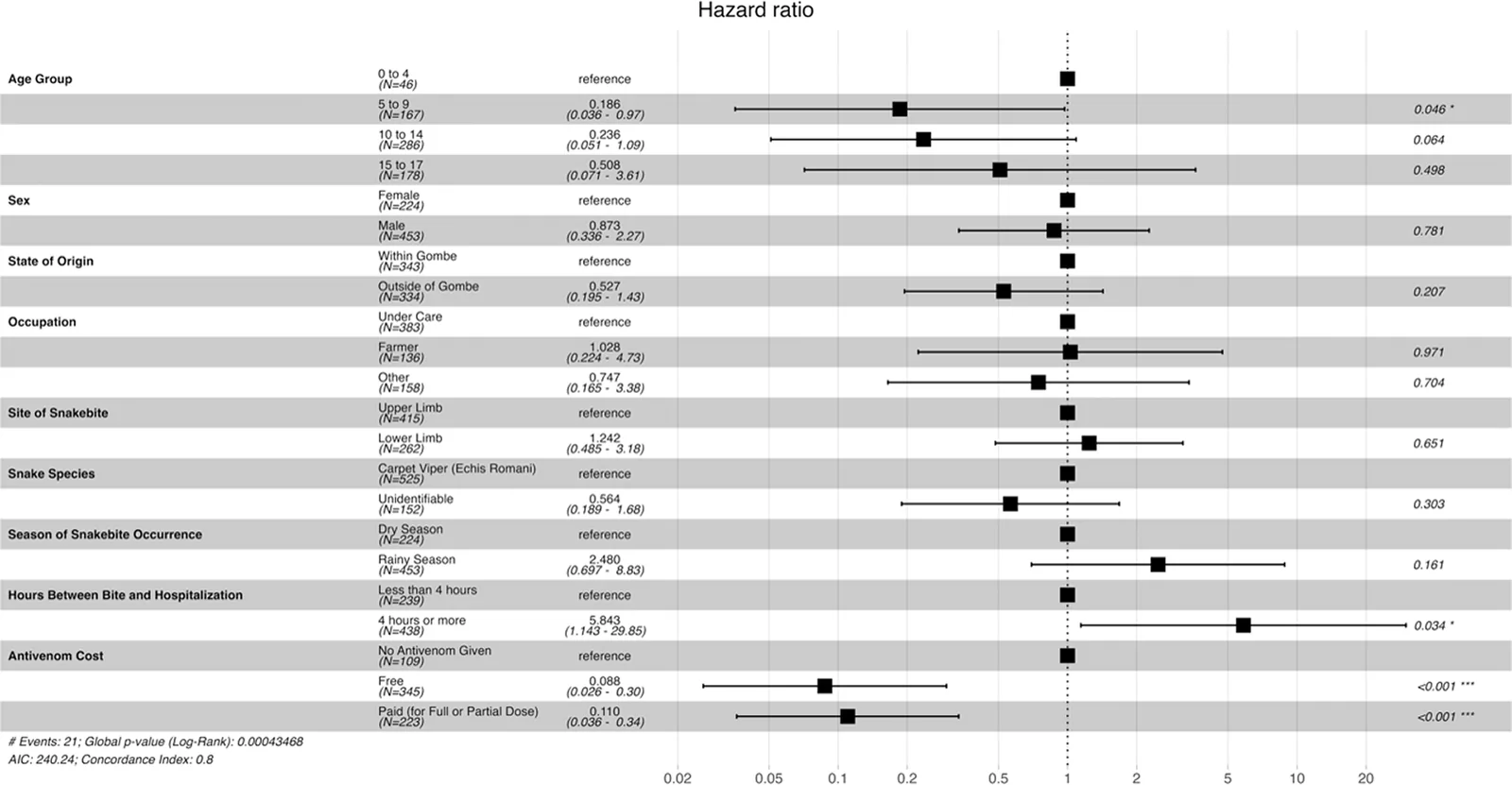
Cox proportional hazards model for survival between snakebite and hospital discharge (antivenom cost).

**Figure 4.**
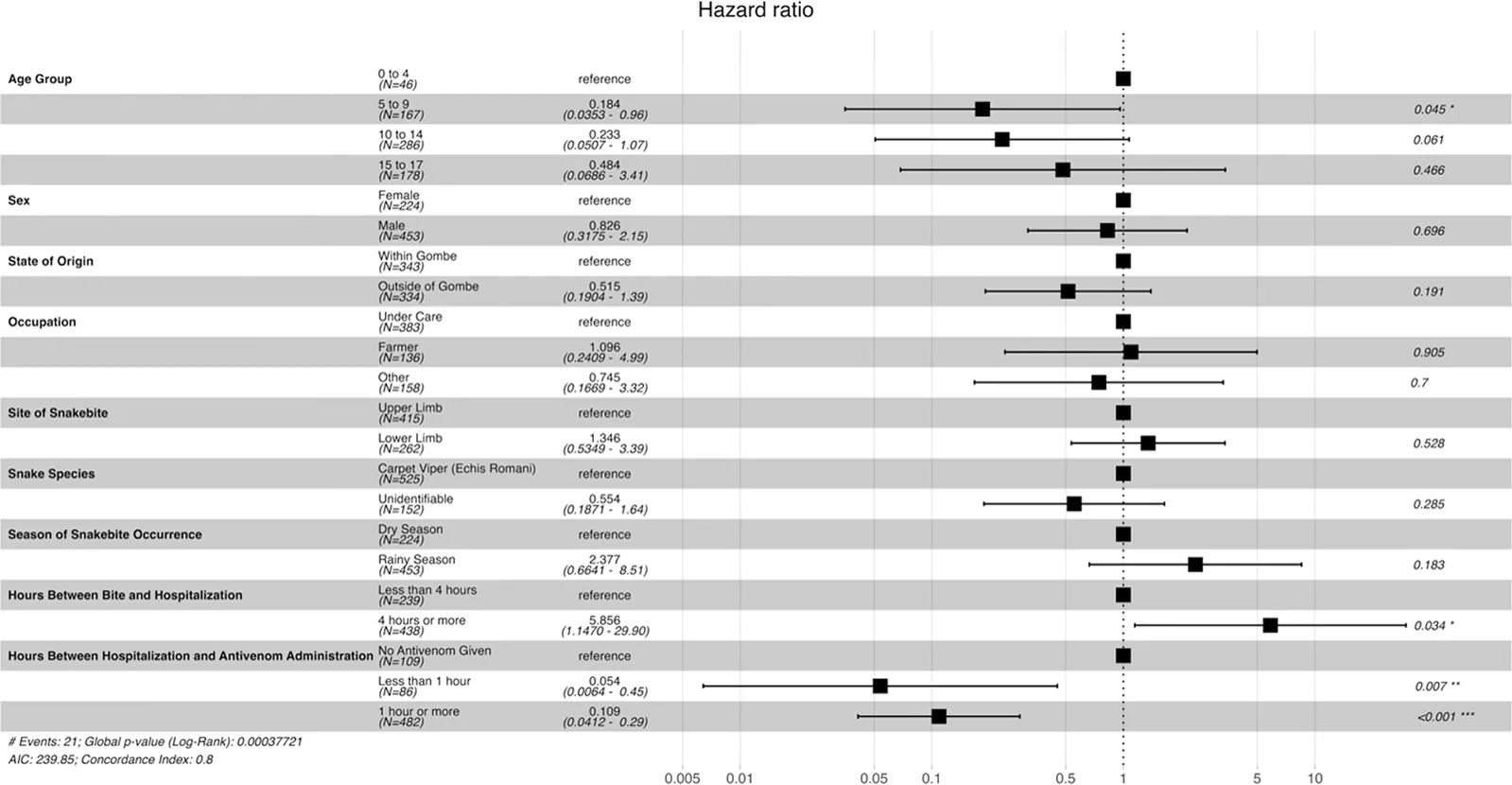
Cox proportional hazards model for survival between snakebite and hospital discharge (time to antivenom administration).

Subsequent Cox proportional hazards modelling revealed important predictors of survival, controlling for confounding factors. Because there was multicollinearity between the three antivenom variables (namely, antivenom administration, antivenom cost, and time to antivenom administration), we opted for three different Cox proportional hazards models, each with one of these variables. Figures 2- 4 describe results for these three models.

In the first model, the time between bite and hospitalization significantly impacted survival. Those who took four hours or more to arrive at the hospital after being bitten were more likely to die at hospital discharge compared to those who arrived in less than four hours (hazard ratio (HR) = 5.87, 95% CI = 1.15-29.95). Further, those who were not given antivenom were more likely to die at hospital discharge than those who were given antivenom (HR = 10.07, 95% CI = 3.89-26.07). Finally, being part of the 5 to 9 age group appeared protective against death (HR = 0.19, 95% CI = 0.04-0.98), compared to the 0 to 4 age group ( Figure 2).

In the second model, the directionality of the association between variables significantly associated with the outcome remained unchanged. Like in the first model, those who took four hours or more to arrive at the hospital after being bitten were more likely to die at hospital discharge compared to those who arrived in less than four hours (HR = 5.84, 95% CI = 1.14-29.85). Additionally, those in the 5 to 9 year-old age group were less likely to die (HR = 0.19, 95% CI 0.04-0.97) compared to those in the 0 to 4 year-old age group. Finally, those who were given free antivenom or paid antivenom were less likely to die at hospital discharge than those were not given antivenom at all (HR = 0.09, 95% CI = 0.03-0.30 for free antivenom; HR = 0.11, 95% CI = 0.04-0.34 for paid antivenom) ( Figure 3).

Finally, in the third model, like in the first and second models, those who took four hours or more to arrive at hospital after being bitten were more likely to die at hospital discharge compared to those who arrived in less than four hours (HR = 5.86, 95% CI = 1.15-29.90). Similarly, patients in the 5 to 9 age group had better survival compared to the 0 to 4 age group (HR = 0.18, 95% CI 0.04-0.96). Further, those who were given antivenom within one hour or after one hour or more were less likely to die at hospital discharge than those were not given antivenom at all (HR = 0.05, 95% CI = 0.01-0.45 for antivenom in less than one hour; HR = 0.11, 95% CI = 0.04-0.29 for paid antivenom) ( Figure 4).

Similar Cox proportional hazards models were created using the time period from hospital arrival to discharge (Figures S3-S5). Two variables, namely age and occupation, were removed from these models due to a violation of the proportional hazards assumption. The significance and directionality of the relationships between time from bite to hospitalization and the three antivenom variables remained consistent in these models. Notably, Cox proportional hazards models were not generated for the time from antivenom administration to hospital discharge due to numerous violations of the proportional hazards assumptions, which prevented the models from converging.

## Discussion

This study presents survival data from 723 paediatric snakebite cases managed at SBTRH between January and December 2024. While most paediatric patients survived their envenomation (97%), there were key factors significantly associated with the likelihood of death. Most notably, we found that paediatric patients who took four hours or more to arrive at the hospital after being bitten were more likely to die in hospital compared to those who arrived in less than four hours, and those who were not given antivenom were more likely to die in hospital than those who were given antivenom.

These findings align with existing literature emphasizing the importance of rapid access to medical care following envenomation ( Abdullahi et al., 2022; Hamman et al., 2025c, 2025d; Iliyasu et al., 2023b; Wood, 2023). Delays in presentation may reflect barriers such as poor transportation infrastructure, reliance on traditional healers, low awareness of the urgency of treatment, and socioeconomic constraints **,** particularly in rural settings **.** Such findings underscore the need for strengthened community education, improved referral systems, and investment in emergency transport infrastructure to reduce time to care.

Antivenom administration was also strongly associated with improved survival across all analyses – a finding that is echoed in the literature. Indeed, over the years, antivenom has been considered the cornerstone of envenomation treatment ( Gamulin et al., 2023; Gutiérrez et al., 2011; Matlani, 2024; Uko et al., 2024), and these results reinforce its essential role in reducing mortality. Notably, patients who did not receive antivenom at our institution were typically affected by stockouts, limited supply, or financial barriers **,** underscoring persistent access challenges. Log-rank tests further confirmed these associations ( *p* < 0.001), consistently showing higher cumulative survival among those who received antivenom, regardless of timing or cost. These findings highlight the urgent need for policies that ensure timely, universal, and affordable access to antivenom, particularly in high-burden, resource-limited settings. By employing survival analysis rather than logistic regression as used in our previous study ( Hamman et al., 2025a), this work provides a time-sensitive understanding of the protective effect of antivenom, offering insights that cannot be obtained from non-time-dependent models.

Age group also demonstrated a significant relationship with survival. Patients aged 5 to 9 had a significantly reduced hazard of death compared to those aged 0 to 4, suggesting that very young children may be more vulnerable to the systemic effects of venom due to smaller body size or differences in physiological response ( Iliyasu et al., 2023b, 2023a; Sankar et al., 2013; Zdenek et al., 2023). This pattern is consistent with evidence suggesting that paediatric patients often deteriorate more rapidly than adults following envenomation, highlighting the value of age-specific survival analyses. However, this warrants further investigation. Targeted interventions and heightened clinical vigilance may therefore be necessary for very young children in areas endemic to snakebites.

Similarly, while sex, occupation, and site of bite were not significantly associated with survival in this cohort, the observed trends still warrant further exploration in larger or multicenter studies to confirm or refute these patterns. In addition, some covariates, such as age and occupation, had to be excluded from the cox proportional hazards models evaluating survival from admission to discharge due to violation of the proportional hazards assumptions. This limitation may have constrained the interpretation of certain subgroup effects in that time frame. 

Seasonality was another key factor associated with survival. In descriptive analyses, patients bitten during the rainy season had worse outcomes compared to those bitten in the dry season (p = 0.045), with an average case fatality rate of 3.5% during the rainy months compared to 1.8% in the dry season – a finding that may reflect increased snake activity, heightened exposure due to farming, or environmental conditions that delay access to care. Similar seasonal patterns have been observed in other regional and global snakebite studies ( Abdullahi et al., 2022; Bravo-Vega et al., 2022; Mohapatra et al., 2011). These findings suggest the need for targeted seasonal preparedness and resource planning, such as stocking antivenom and conducting community education during the high-risk months.

Interestingly, snake species identification was not significantly associated with survival despite the fact that most patients (77%) were bitten by saw-scaled/carpet vipers ( *Echis romani*), a species known for its potent hemotoxic venom ( Baleela et al., 2025; Tijani et al., 2024; Warrell et al., 1977). Despite this, statistical analysis revealed no significant difference in survival based on snake species (p = 0.831), suggesting that species identification alone may not be a reliable predictor of outcome in this context. Of note, an important consideration is also species-specific effectiveness of antivenoms. Echitab, which is the primary antivenom used at SBTRH, has been shown to be effective against saw-scaled/carpet vipers ( Abubakar et al., 2010). While species identification at SBTRH is robust, as in previous studies ( Hamman et al., 2025c, 2025a, 2025b), a proportion of patients were unable to identify the snake species. This highlights the need for improved community education on the importance of snake identification, which can aid in guiding appropriate antivenom use and potentially improve treatment outcomes.

Notably, no covariates were found to be significantly associated with the cumulative survival probability from the time of antivenom administration to hospital discharge. This may suggest that, once antivenom is administered, a patient’s likelihood of survival is largely established. Alternatively, the absence of significant associations may reflect the limited statistical power within this subgroup of patients who received antivenom. Regardless, the finding underscores that a critical window for reducing mortality lies in the period preceding antivenom administration, highlighting the importance of early intervention and rapid access to treatment.

In addition to descriptive and survival analyses, this study leveraged Cox proportional hazards modeling to identify adjusted predictors of mortality. Although all models consistently showed the protective effect of early hospital presentation and antivenom access, multicollinearity among antivenom-related variables limited the interpretability when all three models were included simultaneously. This challenge highlights the statistical complexity of modeling closely related treatment variables, especially when they reflect both clinical decision-making and patient severity.

To ensure full transparency, we acknowledge that the dataset used in this study has been previously described in a separate publication. However, the current analysis addresses a fundamentally different research question predicting in-hospital mortality using survival methods and thereby enables a more detailed and time-sensitive understanding of mortality risk ( Lee, 2023). These insights could not be obtained from the earlier descriptive analysis or logistic regression modelling and therefore represent an important and original contribution.

Our study responds to the growing recognition of the need for targeted paediatric snakebite research by addressing key methodological limitations in the existing literature. Indeed, most existing studies to date rely on simple descriptive or cross-sectional analyses, which fail to account for when deaths occur or how risk evolves over time.

While our study demonstrates the potential for advanced statistical modeling in predicting snakebite outcomes, it also reveals critical limitations. Prominent among these is the single-centre nature of the data, which may limit generalizability to other regions with differing health infrastructure, snake species prevalence, and cultural practices. Additionally, despite using a relatively large paediatric sample for snakebite research, the number of deaths (n = 21) was small, limiting statistical power. Furthermore, certain potentially influential variables such as envenomation severity scores, presence of systemic bleeding or neurological symptoms, and initial vital signs were not available in the dataset. These factors could further refine predictive models and clinical algorithms. Limited events and unmeasured confounding are common disadvantages of retrospective data; as well, retrospective data is more prone to bias, missing information, and has lower quality than prospective data. Nonetheless, SBTRH keeps meticulous records and most variables included in analyses had complete data for patients in this study.

Future studies should focus on multicentre collaborations that standardize data collection and reporting. Inclusion of clinical markers of severity and real-time physiological data would enhance the development of more robust prognostic tools. Moreover, integrating these tools into clinical workflows, such as electronic decision-support systems, has the potential to improve outcomes through timely triage and intervention.

## Conclusions

Overall, this study identifies delayed hospital presentation, lack of antivenom administration, and, potentially, very young age as key predictors of in-hospital mortality among paediatric snakebite patients in Northeastern Nigeria. By applying a survival analysis framework and focusing specifically on the timing of mortality, this study provides novel and clinically meaningful insights that extend beyond earlier epidemiologic findings and emphasize the importance of early access to care. These findings underscore the need for urgent public health interventions, including the strengthening of antivenom supply chains, eliminating financial barriers to treatment, and the implementation of targeted educational campaigns aimed at early care-seeking behaviour.

## Data Availability

Data are available under the terms of the Creative Commons Attribution 4.0 International license (CC-BY 4.0); a deidentified subset of the full dataset (677 of 723 patients that were included in survival analyses) is included in the Oxford University Research Archive ( https://dx.doi.org/10.5287/ora-v0zj27jge) ( Hamman et al., 2026). The full dataset of 723 patients is not provided due to low cell counts for certain variables, which could potentially identify individual patients.
