# Supplementary material for: Predictors of In-Hospital Mortality Among Paediatric Snakebite Patients in Northeastern Nigeria: A Survival Analysis Study
Source: Wellcome Open Res. 2026 Jul 6;11:244. Originally published 2026 Apr 28. [Version 2] doi: 10.12688/wellcomeopenres.26196.2 (PMC13555079; doi:10.12688/wellcomeopenres.26196.2)
Supplement: Peer review history

# Reviewer report: Reviewer response for version 2

**DOI:** 10.21956/wellcomeopenres.29895.r160116

**Published:** 2026-07-22

**Authors:** Abdullah Abu Sayeed

**Affiliations:** 1 Chittagong Medical College, Chittagong, Chittagong Division, Bangladesh

It's now in a good shape for indexing.

Is the work clearly and accurately presented and does it cite the current literature?

Yes

If applicable, is the statistical analysis and its interpretation appropriate?

Yes

Are all the source data underlying the results available to ensure full reproducibility?

Partly

Is the study design appropriate and is the work technically sound?

Yes

Are the conclusions drawn adequately supported by the results?

Partly

Are sufficient details of methods and analysis provided to allow replication by others?

Yes

Reviewer Expertise:

NA

I confirm that I have read this submission and believe that I have an appropriate level of expertise to confirm that it is of an acceptable scientific standard.

# Reviewer report: Reviewer response for version 2

**DOI:** 10.21956/wellcomeopenres.29895.r160115

**Published:** 2026-07-22

**Authors:** Jaideep C Menon, Anu Suresh

**Affiliations:** 1 Amrita Vishwa Vidyapeetham, Kochi, Kerala, India

Thank you for the revised manuscript and the detailed point-by-point responses. The authors have satisfactorily addressed the concerns raised in my previous review. The revisions have improved the clarity of the manuscript, particularly regarding the interpretation of antivenom administration, the discussion on snake identification, and the acknowledgment of limitations related to unavailable clinical outcome data. I have no further major comments and recommend acceptance of the manuscript.

Is the work clearly and accurately presented and does it cite the current literature?

Yes

If applicable, is the statistical analysis and its interpretation appropriate?

Yes

Are all the source data underlying the results available to ensure full reproducibility?

Yes

Is the study design appropriate and is the work technically sound?

Yes

Are the conclusions drawn adequately supported by the results?

Yes

Are sufficient details of methods and analysis provided to allow replication by others?

Yes

Reviewer Expertise:

non-communicable diseases, cardiovascular diseases, population health sciences, venomous snakebite

We confirm that we have read this submission and believe that we have an appropriate level of expertise to confirm that it is of an acceptable scientific standard.

# Reviewer report: Reviewer response for version 1

**DOI:** 10.21956/wellcomeopenres.28854.r156189

**Published:** 2026-06-13

**Authors:** Abdullah Abu Sayeed

**Affiliations:** 1 Chittagong Medical College, Chittagong, Chittagong Division, Bangladesh

This is a well-written, retrospective cohort study that utilizes time-to-event statistical methods (Kaplan-Meier estimation and Cox proportional hazards models) to evaluate predictors of in-hospital mortality among 723 pediatric snakebite patients in Northeastern Nigeria. By applying a survival analysis framework rather than standard logistic regression, the authors successfully capture vital temporal dimensions of pediatric snakebite mortality. The manuscript addresses a notable gap in regional literature, highlighting that delayed hospital presentation (>4 hours) and a lack of antivenom significantly increase the risk of death. While the dataset is robust and the methodology is technically sound, several areas regarding data reporting, confounding interpretations, and clinical context require clarification before full approval.

 Sociodemographic Anomalies in Table 1  Pediatric Housewives: In Table 1, under the "Occupation" category, the authors list 21 patients as "House Wife". Given that the study population is strictly defined as children aged 0 through 17 years, the presence of married minors warrants explicit contextualization regarding local socioeconomic customs, or verification to ensure it is not a data entry error. Percentage Reporting: The percentages reported across several rows in Table 1 do not cleanly sum to 100%. The authors must carefully audit the descriptive statistics to ensure mathematical consistency throughout the categorical data presentation.

 Reporting of Demographic Significance  In the third paragraph of the Results section, the authors omit a meaningful finding shown in Table 1: the statistically significant association between a patient's state of origin and survival status (p = 0.024). Notably, patients from Borno State experienced a substantially higher raw mortality rate (12\%) compared to other regions. This demographic variable needs to be integrated into the narrative results and briefly explored in the Discussion (e.g., assessing whether it functions as a proxy for structural barriers, travel distance, or regional delays).

 Confounding Interpretation of "Antivenom Cost" * The interpretation of "Antivenom Cost" (Model 2, Figure 3) as an independent predictor of survival is potentially misleading. In the multivariable analysis, the chosen reference category consists of patients who did not receive antivenom at all. Consequently, the drastically reduced hazard ratios for both the "Free" (HR = 0.09) and "Paid" (HR = 0.11) cohorts simply reflect the profound clinical benefit of receiving the therapeutic agent, rather than the impact of financial mechanics.  To evaluate the true independent effect of cost on survival, a direct comparison between patients receiving free versus paid antivenom is required. A Fisher's exact test isolated to these two treated groups reveals no statistically significant difference in survival (p = 0.26). The narrative should be revised to avoid implying that the pricing model itself directly altered clinical survival outcomes. Practical Implications of Snake Species Identification  In the Discussion, the authors emphasize the need for community education on snake species identification to "aid in guiding appropriate antivenom use." However, as noted in the manuscript, the primary therapeutic countermeasure used at SBTRH is Echitab—a polyvalent antivenom highly effective against the predominant regional vipers. Because antivenom administration in low-resource syndromic workflows is properly guided by clinical signs of envenomation rather than definitive herpetological identification, the authors should clarify the actual clinical utility of species identification in this specific treatment pathway.

 Clinical Utility of Post-Antivenom Metrics  The authors note that "no covariates were significantly associated with cumulative survival probability from the time of antivenom administration to hospital discharge." Evaluating simple discharge velocity after therapeutic administration offers limited prognostic value. To increase the clinical relevance of the post-admission phase, the study would benefit from tracking intermediate, highly informative in-hospital complications—such as the development of acute kidney injury (AKI), severe coagulopathy, neurotoxic shock, or the subsequent requirement for mechanical ventilation or intensive care. If these clinical markers are unavailable due to retrospective limitations, this should be explicitly listed as a constraint. In Conclusion :The core survival analysis framework provides highly actionable evidence that early healthcare seeking and protected supply chains are essential to pediatric survival. Addressing these data presentation issues and refining the clinical interpretations will significantly strengthen the impact of this manuscript.

Is the work clearly and accurately presented and does it cite the current literature?

Yes

If applicable, is the statistical analysis and its interpretation appropriate?

Yes

Are all the source data underlying the results available to ensure full reproducibility?

Partly

Is the study design appropriate and is the work technically sound?

Yes

Are the conclusions drawn adequately supported by the results?

Partly

Are sufficient details of methods and analysis provided to allow replication by others?

Yes

Reviewer Expertise:

Infectious Diseases, Malaria, Dengue and Snakebite

I confirm that I have read this submission and believe that I have an appropriate level of expertise to confirm that it is of an acceptable scientific standard, however I have significant reservations, as outlined above.

# Response

**Published:** 2026-06-26

**Authors:** Aashna Uppal

**Affiliations:** Nuffield Department of Medicine, University of Oxford Nuffield Department of Medicine, Oxford, England, UK

**Revisions based on Reviewer 2**   This is a well-written, retrospective cohort study that utilizes time-to-event statistical methods (Kaplan-Meier estimation and Cox proportional hazards models) to evaluate predictors of in-hospital mortality among 723 pediatric snakebite patients in Northeastern Nigeria. By applying a survival analysis framework rather than standard logistic regression, the authors successfully capture vital temporal dimensions of pediatric snakebite mortality. The manuscript addresses a notable gap in regional literature, highlighting that delayed hospital presentation (>4 hours) and a lack of antivenom significantly increase the risk of death. While the dataset is robust and the methodology is technically sound, several areas regarding data reporting, confounding interpretations, and clinical context require clarification before full approval.  

1. Sociodemographic Anomalies in Table 1 Pediatric Housewives: In Table 1, under the "Occupation" category, the authors list 21 patients as "House Wife". Given that the study population is strictly defined as children aged 0 through 17 years, the presence of married minors warrants explicit contextualization regarding local socioeconomic customs, or verification to ensure it is not a data entry error.

**  Response: **Indeed, the youngest age of marriage among Fulani women is 11-12 years old in this region, so it is possible that we find housewives among this paediatric population. We have added a footnote to this effect under Table 1.    

1. Percentage Reporting: The percentages reported across several rows in Table 1 do not cleanly sum to 100%. The authors must carefully audit the descriptive statistics to ensure mathematical consistency throughout the categorical data presentation.

 
**  Response: ** We have gone through Table 1 and have ensured that all rows add up to 100%.  

1. Reporting of Demographic Significance In the third paragraph of the Results section, the authors omit a meaningful finding shown in Table 1: the statistically significant association between a patient's state of origin and survival status (p = 0.024). Notably, patients from Borno State experienced a substantially higher raw mortality rate (12\%) compared to other regions. This demographic variable needs to be integrated into the narrative results and briefly explored in the Discussion (e.g., assessing whether it functions as a proxy for structural barriers, travel distance, or regional delays).

**  Response: ** We have noted in the results section that “state of origin, which often reflects distance to hospital (p = 0.024), were all significantly associated with survival”. We have also added to our discussion: “The observed association between state of origin and survival may similarly reflect geographic and structural barriers to timely access to care, particularly for patients travelling longer distances to reach SBTRH.”  

1. Confounding Interpretation of "Antivenom Cost" * The interpretation of "Antivenom Cost" (Model 2, Figure 3) as an independent predictor of survival is potentially misleading. In the multivariable analysis, the chosen reference category consists of patients who did not receive antivenom at all. Consequently, the drastically reduced hazard ratios for both the "Free" (HR = 0.09) and "Paid" (HR = 0.11) cohorts simply reflect the profound clinical benefit of receiving the therapeutic agent, rather than the impact of financial mechanics.  To evaluate the true independent effect of cost on survival, a direct comparison between patients receiving free versus paid antivenom is required. A Fisher's exact test isolated to these two treated groups reveals no statistically significant difference in survival (p = 0.26). The narrative should be revised to avoid implying that the pricing model itself directly altered clinical survival outcomes.

**  Response: ** We have considered a subgroup analysis among those who received antivenom. We have added the following text in the results: “We also considered a sub-group analysis with only those who received antivenom to explore the relationship that both cost and timing have with mortality. Paying for antivenom and delaying its administration more than one hour indeed increased odds of mortality among those who received antivenom, but estimates were not statistically significant (Fig S3).” We have also added to our discussion: “However, subgroup analysis restricted to patients who received antivenom found no statistically significant association between whether antivenom was provided free of charge or paid for and mortality suggesting that the observed effect in the primary analysis reflects the benefit of receiving antivenom rather than the payment mechanism itself.”  

1. Practical Implications of Snake Species Identification  In the Discussion, the authors emphasize the need for community education on snake species identification to "aid in guiding appropriate antivenom use." However, as noted in the manuscript, the primary therapeutic countermeasure used at SBTRH is Echitab—a polyvalent antivenom highly effective against the predominant regional vipers. Because antivenom administration in low-resource syndromic workflows is properly guided by clinical signs of envenomation rather than definitive herpetological identification, the authors should clarify the actual clinical utility of species identification in this specific treatment pathway.

**  Response: ** We have expanded our discussion to say: “While species identification at SBTRH is robust, as in previous studies (Hamman et al., 2025c, 2025a, 2025b), a proportion of patients were unable to identify the offending snake. Although antivenom administration at SBTRH is guided primarily by clinical evidence of envenomation rather than species identification, accurate species identification remains important for epidemiological surveillance, monitoring species distribution, evaluating antivenom effectiveness and informing future public health strategies. Furthermore, because our cohort was dominated by the three most commonly encountered snake species, conclusions regarding the association between snake species and survival should be interpreted cautiously and may not be generalizable to less frequently encountered species. Continued efforts to improve snake identification therefore remain important for research and surveillance, even when immediate clinical management is guided by syndromic assessment.”  

1. Clinical Utility of Post-Antivenom Metrics  The authors note that "no covariates were significantly associated with cumulative survival probability from the time of antivenom administration to hospital discharge." Evaluating simple discharge velocity after therapeutic administration offers limited prognostic value. To increase the clinical relevance of the post-admission phase, the study would benefit from tracking intermediate, highly informative in-hospital complications—such as the development of acute kidney injury (AKI), severe coagulopathy, neurotoxic shock, or the subsequent requirement for mechanical ventilation or intensive care. If these clinical markers are unavailable due to retrospective limitations, this should be explicitly listed as a constraint.

**  Response: ** We have added to our discussion: “Additionally, detailed post-antivenom clinical outcome measures were not consistently recorded and therefore could not be evaluated. Important intermediate in-hospital complications, such as acute kidney injury (AKI), severe coagulopathy, neurotoxic shock, or the subsequent need for mechanical ventilation or intensive care, may provide more clinically informative indicators of patient progression after antivenom administration than discharge outcomes alone. The absence of these data limited our ability to assess the prognostic significance of post-antivenom clinical trajectories and may partially explain why no covariates were significantly associated with cumulative survival probability from the time of antivenom administration to hospital discharge”  

1. In Conclusion: The core survival analysis framework provides highly actionable evidence that early healthcare seeking, and protected supply chains are essential to pediatric survival. Addressing these data presentation issues and refining the clinical interpretations will significantly strengthen the impact of this manuscript.

**  Response: ** Thank you for taking the time to review this paper!

# Reviewer report: Reviewer response for version 1

**DOI:** 10.21956/wellcomeopenres.28854.r155020

**Published:** 2026-06-03

**Authors:** Jaideep C Menon

**Affiliations:** 1 Amrita Vishwa Vidyapeetham, Kochi, Kerala, India

I read the manuscript titled 'Predictors of In-Hospital Mortality Among Paediatric Snakebite Patients in Northeastern Nigeria: A Survival Analysis Study' with considerable interest. I have the following suggestions and clarifications to make

 - one of the reasons for poorer outcomes in children includes the short circulation time and the fact that children panic and often run when bitten, facilitating venom absorption.

 - in the table the authors list 21 victims as housewives? this from a pediatric population?

 - 

1. In Table 1, the percentages reported across several rows do not sum to 100%, suggesting potential errors in percentage calculation or reporting. The authors are advised to carefully verify the calculations and ensure consistency in the presentation of categorical data.
2. Table 1 demonstrates a statistically significant association between patients’ state of origin and survival status (p = 0.024). However, this finding has not been adequately described in the Results section (third paragraph). The authors should incorporate and discuss this observation in the corresponding narrative results.
3. The interpretation regarding “antivenom cost” as a significant predictor of survival appears potentially misleading. In the analyses, the reference category consisted of patients who did not receive antivenom, while comparisons were made separately with patients receiving free or paid antivenom. Therefore, the observed association likely reflects the effect of antivenom administration itself rather than whether the antivenom was free or paid. A direct comparison between patients receiving free versus paid antivenom would be more appropriate to evaluate the effect of cost on survival. Based on the Fisher’s exact test performed between these two groups, no statistically significant association with survival was observed (p = 0.26).
4. In the Discussion section, the authors mention that a proportion of patients were unable to identify the snake species and emphasize the importance of snake identification for guiding treatment. This statement may require further clarification. Since Echitab is a polyvalent antivenom and antivenom administration is generally guided by clinical evidence of envenomation rather than definitive species identification, the practical implications of inability to identify the snake species on treatment decisions and outcomes should be elaborated more clearly.
5. The statement that “no covariates were significantly associated with cumulative survival probability from the time of antivenom administration to hospital discharge” may have limited clinical relevance as currently presented. Instead of focusing primarily on duration from antivenom administration to discharge, the authors may consider evaluating clinically meaningful in-hospital outcomes or complications such as acute kidney injury, requirement for mechanical ventilation, coagulopathy, shock, or intensive care admission. These parameters may provide more informative insights into disease severity and patient prognosis following antivenom administration.
6. In Table 1, the percentages reported across several rows do not sum to 100%, suggesting potential errors in percentage calculation or reporting. The authors are advised to carefully verify the calculations and ensure consistency in the presentation of categorical data.
7. Table 1 demonstrates a statistically significant association between patients’ state of origin and survival status (p = 0.024). However, this finding has not been adequately described in the Results section (third paragraph). The authors should incorporate and discuss this observation in the corresponding narrative results.
8. The interpretation regarding “antivenom cost” as a significant predictor of survival appears potentially misleading. In the analyses, the reference category consisted of patients who did not receive antivenom, while comparisons were made separately with patients receiving free or paid antivenom. Therefore, the observed association likely reflects the effect of antivenom administration itself rather than whether the antivenom was free or paid. A direct comparison between patients receiving free versus paid antivenom would be more appropriate to evaluate the effect of cost on survival. Based on the Fisher’s exact test performed between these two groups, no statistically significant association with survival was observed (p = 0.26).
9. In the Discussion section, the authors mention that a proportion of patients were unable to identify the snake species and emphasize the importance of snake identification for guiding treatment. This statement may require further clarification. Since Echitab is a polyvalent antivenom and antivenom administration is generally guided by clinical evidence of envenomation rather than definitive species identification, the practical implications of inability to identify the snake species on treatment decisions and outcomes should be elaborated more clearly.
- The statement that “no covariates were significantly associated with cumulative survival probability from the time of antivenom administration to hospital discharge” may have limited clinical relevance as currently presented. Instead of focusing primarily on duration from antivenom administration to discharge, the authors may consider evaluating clinically meaningful in-hospital outcomes or complications such as acute kidney injury, requirement for mechanical ventilation, coagulopathy, shock, or intensive care admission. These parameters may provide more informative insights into disease severity and patient prognosis following antivenom administration. 

Is the work clearly and accurately presented and does it cite the current literature?

Yes

If applicable, is the statistical analysis and its interpretation appropriate?

Yes

Are all the source data underlying the results available to ensure full reproducibility?

Yes

Is the study design appropriate and is the work technically sound?

Yes

Are the conclusions drawn adequately supported by the results?

Yes

Are sufficient details of methods and analysis provided to allow replication by others?

Yes

Reviewer Expertise:

non-communicable diseases, cardiovascular diseases, population health sciences, venomous snakebite

I confirm that I have read this submission and believe that I have an appropriate level of expertise to confirm that it is of an acceptable scientific standard, however I have significant reservations, as outlined above.

# Response

**Published:** 2026-06-26

**Authors:** Aashna Uppal

**Affiliations:** Nuffield Department of Medicine, University of Oxford Nuffield Department of Medicine, Oxford, England, UK

**Revisions based on Reviewer 1**   I read the manuscript titled 'Predictors of In-Hospital Mortality Among Paediatric Snakebite Patients in Northeastern Nigeria: A Survival Analysis Study' with considerable interest. I have the following suggestions and clarifications to make   - one of the reasons for poorer outcomes in children includes the short circulation time and the fact that children panic and often run when bitten, facilitating venom absorption. Response: We have added this to the introduction, lines 71-73.   - in the table the authors list 21 victims as housewives? this from a pediatric population? Response: Indeed, the youngest age of marriage among Fulani women is 11-12 years old in this region, so it is possible that we find housewives among this paediatric population. We have added a footnote to this effect under Table 1.  

1. In Table 1, the percentages reported across several rows do not sum to 100%, suggesting potential errors in percentage calculation or reporting. The authors are advised to carefully verify the calculations and ensure consistency in the presentation of categorical data.

**Response: **We have gone through Table 1 and have ensured that all rows add up to 100%.

1. Table 1 demonstrates a statistically significant association between patients’ state of origin and survival status (p = 0.024). However, this finding has not been adequately described in the Results section (third paragraph). The authors should incorporate and discuss this observation in the corresponding narrative results.

**Response:** We’ve noted in our results that state of origin often reflects distance to hospital.

1. The interpretation regarding “antivenom cost” as a significant predictor of survival appears potentially misleading. In the analyses, the reference category consisted of patients who did not receive antivenom, while comparisons were made separately with patients receiving free or paid antivenom. Therefore, the observed association likely reflects the effect of antivenom administration itself rather than whether the antivenom was free or paid. A direct comparison between patients receiving free versus paid antivenom would be more appropriate to evaluate the effect of cost on survival. Based on the Fisher’s exact test performed between these two groups, no statistically significant association with survival was observed (p = 0.26).

**Response: **We’ve added to our analyses accordingly: “We also considered a sub-group analysis with only those who received antivenom to explore the relationship that both cost and timing have with mortality. Paying for antivenom and delaying its administration more than one hour indeed increased odds of mortality among those who received antivenom, but estimates were not statistically significant (Fig S3).”

1. In the Discussion section, the authors mention that a proportion of patients were unable to identify the snake species and emphasize the importance of snake identification for guiding treatment. This statement may require further clarification. Since Echitab is a polyvalent antivenom and antivenom administration is generally guided by clinical evidence of envenomation rather than definitive species identification, the practical implications of inability to identify the snake species on treatment decisions and outcomes should be elaborated more clearly.

**Response:** We have added the following lines to the discussion: “However, antivenom administration at SBTRH is guided primarily by clinical evidence of envenomation, including abnormalities on the 20-minute whole blood clotting test (20WBCT) and other clinical signs, rather than snake identification alone. The use of a polyvalent antivenom such as Echitab also helps ensure appropriate treatment when the offending species cannot be confirmed. Thus, because treatment is guided primarily by the 20-minute whole blood clotting test and clinical signs of envenomation, inability to identify the snake species does not usually alter initial management.”

1. The statement that “no covariates were significantly associated with cumulative survival probability from the time of antivenom administration to hospital discharge” may have limited clinical relevance as currently presented. Instead of focusing primarily on duration from antivenom administration to discharge, the authors may consider evaluating clinically meaningful in-hospital outcomes or complications such as acute kidney injury, requirement for mechanical ventilation, coagulopathy, shock, or intensive care admission. These parameters may provide more informative insights into disease severity and patient prognosis following antivenom administration.

**Response:** These other complications are not consistently reported, so we focused on mortality. We have added the following to the discussion: “Additionally, clinically meaningful post-antivenom outcomes such as acute kidney injury, coagulopathy, shock, intensive care admission or mechanical ventilation were not consistently documented in the retrospective dataset and therefore could not be evaluated, although these measures may provide more informative insights into patient prognosis following antivenom administration”.
